# Braithwaite’s Retrospect of Practical Medicine

**Published:** 1846-11

**Authors:** 


ART. XI.—*Braithwaite's Retrospect of Practical Medicine.* New York: Daniel Adee.

The most conclusive evidence we could give of our high estimate of the excellence of this compilation, is the frequent appearance of its matter under our head of "Selections." It is, beyond all doubt, an abstract of the very highest value, and has proved acceptable to the profession in an uncommon degree.

